# Ultrasound-Assisted Natural Deep Eutectic Solvents Extraction of Bilberry Anthocyanins: Optimization, Bioactivities, and Storage Stability

**DOI:** 10.3390/plants11202680

**Published:** 2022-10-12

**Authors:** Miloš S. Jovanović, Nemanja Krgović, Jelena Živković, Tatjana Stević, Gordana Zdunić, Dubravka Bigović, Katarina Šavikin

**Affiliations:** 1 Department of Pharmacy, Faculty of Medicine, University of Niš, Boulevard Dr. Zorana Đinđića 81, 18000 Niš, Serbia; 2 Institute for Medicinal Plants Research “Dr. Josif Pančić”, Tadeuša Košćuška 1, 11000 Belgrade, Serbia

**Keywords:** *Vaccinium myrtillus*, anthocyanins, natural deep eutectic solvents, ultrasound-assisted extraction, green extractions, stability

## Abstract

Bilberry fruits (*Vaccinium myrtillus* L.) are one of the richest natural sources of anthocyanins and are widely used due to their pharmacological and nutritional properties. To ensure their maximum application potential, it is necessary to overcome the limitations of conventional extraction solvents and techniques. This study aimed to develop a green method for bilberry anthocyanin extraction using natural deep eutectic solvents (NaDES) integrated with ultrasound-assisted extraction (UAE) in order to define extraction conditions that will prevent decomposition of the anthocyanins or the loss of bioactivity. After a screening of ten different NaDES, choline chloride:sorbitol (1:1) was selected as the most effective. Furthermore, the influence analysis and optimization of the NaDES–UAE extraction conditions were carried out employing response surface methodology. The optimal conditions were found to be an extraction time of 37.63 min, a temperature of 48.38 °C, and 34.79% (*w*/*w*) water in NaDES. The extraction yields of target compounds under optimized extraction conditions were 0.27 mg/g DW of cyanidin-3-*O*-glucoside and 2.12 mg CGE/g DW of TAC. The obtained optimized extract showed promising radical scavenging and antimicrobial activity. A stability study with the optimized extract revealed that refrigerated storage at 4 °C in the dark provided the best anthocyanins preservation. Overall, the developed NaDES-UAE method showed promising application potential and can be considered as a high-efficiency green alternative to conventional anthocyanins extraction methods, enabling the preservation of active ingredients and the bioactivity of extracts.

## 1. Introduction

The native European blueberry (*Vaccinium myrtillus* L., Ericaceae), also known as the bilberry, is one of the most economically important berries in Europe and is highly valued for its nutritional and medicinal properties [1]. According to the European Scientific Cooperative on Phytotherapy-ESCOP monograph [2], indications for the internal use of bilberry fruits are disorders associated with vasculopathy (such as peripheral vascular insufficiency, heavy and painful legs, and eye microcirculation disorders) and non-specific acute diarrhea. Indications for topical application are the treatment of superficial wounds and mild inflammations of the mouth and throat mucous membranes. The medicinal properties of bilberry fruits are attributed to polyphenolic secondary metabolites, which mainly include anthocyanins, tannins, proanthocyanidins, flavonoids, and phenolic acid derivatives [1,2]. The bilberry anthocyanin profile includes fifteen different anthocyanin glycosides formed by linking five aglycones (cyanidin, delphinidin, peonidin, petunidin, and malvidin) and three sugar components (glucose, galactose, and arabinose) [3,4]. Apart from their vital role in the prevention of various diseases, anthocyanins, as water-soluble pigments of vascular flora, are also noteworthy as safe natural colorants [5,6]. The main challenge in the development of formulations containing anthocyanins is their high instability and, thus, their low bioavailability and short shelf life [6]. Their stability is significantly influenced by different factors, such as temperature, light, pH, enzymes, or oxygen. These factors can lead to anthocyanins degradation, thus reducing their activity [7].

Conventional methods for phytochemicals extraction are time- and energy-consuming and often require the use of organic solvents that are not labeled as GRAS (“generally recognized as safe”). Being economically and environmentally undesirable, such methods do not meet the principles of green chemistry. Even if GRAS solvents, such as ethanol or water are used, the resulting extracts are often not immediately ready for use and require additional processing steps [8]. In the last decades, a new class of solvents termed natural deep eutectic solvents (NaDES) has emerged. NaDES have extensively garnered the interest of researchers and earned the epithet “solvents for the 21st century” due to their wide-spectrum applicability. In fact, NaDES are a mixture of primary plant metabolites, such as sugars (glucose, fructose, sucrose, trehalose, maltose), sugar alcohols (sorbitol, glycerol, 1,2-propanediol, xylitol), organic acids (lactic, citric, maleic, malic, oxalic, ascorbic), amino acids (proline, glycine, alanine), and amines (betaine, choline) [9]. The NaDES components play the role of hydrogen bond donors or acceptors and can build an intermolecular hydrogen bond network resulting in the lowering of the mixture melting point and forming the viscous liquid systems of outstanding solubilizing properties [10]. Other favorable properties of the NaDES include simple and inexpensive synthesis, sustainability, non-flammability, non-volatility, biodegradability, and biocompatibility [11].

NaDES-based extraction is a promising, efficient, and eco-friendly approach for the extraction of anthocyanins from various plant matrices [5,12,13,14]. Beyond extraction improvement, NaDES could contribute to solving other challenges related to the development of anthocyanin formulations. Since the components of NaDES are biomolecules that can be found in the daily diet or nutritional supplements, the extracts can be used directly, bypassing the complicated processing steps, such as separation and purification. The components of the NaDES by themselves can exhibit various biological activities, such as antioxidant and antimicrobial [14]. The hydrogen bond network of NaDES can contribute to the stabilization of the anthocyanin under different storage conditions, thereby extending shelf life [13]. Finally, NaDES-based extracts can be used as a strategy to improve the oral bioavailability of anthocyanins [15].

The high viscosity of NaDES leads to a slowdown in mass transfer and reduces the extraction of target compounds from plant matrices. To overcome these restrictions NaDES are often integrated with accelerating extraction techniques, such as ultrasound-assisted extraction (UAE). Namely, ultrasound through the cavitation bubble collapse generates a strong shear force and “hot spots” of extremely high temperature that reduce solvent viscosity but also the fragmentation of plant material and facilitate solvent penetration [16,17]. Additionally, ultrasound disrupts the structure of the plant matrices and intensifies the mass transfer [18]. This contributes to shortening the extraction time and allows extraction at a relatively low temperature, which is advantageous in the case of unstable compounds, such as anthocyanins [14]. A key factor affecting the efficiency of NaDES-based extraction is their viscosity and, to a lesser extent, polarity and acidity [12]. Fortunately, these properties of NaDES can be adjusted as needed depending on the target compound (selecting appropriate extraction technique, selecting components of the mixture, adding water, controlling temperature). Such fine-tuned solvents allow extraction selectivity. Therefore, to ensure extraction efficiency, great attention is paid to the selection of NaDES components and the optimization of the process conditions. Response surface methodology (RSM) is often used for these purposes. RSM is a mathematical tool that enables the influence analysis, prediction, and optimization of extraction conditions [18].

Although great efforts are being made to develop efficient green extraction methods, to our knowledge, there were no studies in which NaDES have been evaluated for bilberry anthocyanin extraction. Thus, this study aimed to develop an innovative eco-friendly method for the extraction of anthocyanins from bilberry fruits using NaDES integrated with the UAE with extraction conditions that will prevent the decomposition of the ingredients or the loss of bioactivity. For this purpose, the extraction efficiency of different choline chloride-based NaDES was examined and the most suitable one was selected. Furthermore, the optimization of the extraction conditions was performed using RSM. Finally, the storage stability and biological activities (antimicrobial and antioxidant) of the optimized extract were estimated.

## 2. Results and Discussion

### 2.1. Screening Analysis of NaDES

As shown in Table 1, the different compositions of the NaDES significantly affected the extraction efficiency of the target compounds. Compared to methanol as a conventional solvent, all NaDES showed lower extraction yields of total anthocyanins content (TAC) but equal or higher yields of cyanidin-3-*O*-glucoside. The extraction efficiency of NaDES ranged from 0.92 ± 0.01 to 2.48 ± 0.02 mg CGE/g DW for TAC and from 0.19 ± 0.01 to 0.29 ± 0.00 mg/g DW for cyanidin-3-*O*-glucoside. The low efficacy of the urea-containing NaDES 10 for both responses can be explained by the known property of anthocyanins that is susceptible to degradation under alkaline conditions. Interestingly, the highest yield of TAC and the lowest yield of cyanidin-3-*O*-glucoside were recorded for the same NaDES 1 solvent containing lactic acid. In fact, NaDES of low pH and viscosity, such as NaDES 1, favor the extraction of polyphenolic compounds [10]. The yield discrepancy between TAC and individual cyanidin-3-*O*-glucosides can be explained by the acid hydrolysis of anthocyanin heterosides on sugar and aglycone units that can occur at elevated temperatures. In solvents of lower viscosity, the mobility of solutes is intensified and thus their stabilizing properties are weakened [12]. On the other hand, other acid-based solvents (NaDES 2–4) showed lower extraction efficiencies for TAC but higher efficiencies for cyanidin-3-*O*-glucoside compared to NaDES 1. This is probably due to the difference in viscosity among NaDES. Choline chloride-based NaDES with citric, malic, and tartaric acid usually show a higher viscosity than lactic acid [10]. The higher viscosity of NaDES is usually correlated with weaker extractability but improved stabilization properties. All considered, there were no clear correlations between extraction efficiency and the acidity behavior of NaDES ingredients. This is consistent with the statement of Dai and co-workers [12] who studied the anthocyanins extraction using different NaDES on the *Catharanthus roseus* peltates plant model.

Previous research has shown that acid-based NaDES are frequently the choice for anthocyanin extraction. However, this study shows that satisfactory extraction efficiency can be achieved using sugars and sugar alcohol-based NaDES. Among them, the mixture with sorbitol (NaDES 7) proved to be the most effective. Considering all the screened solvents, NaDES 7 was moderately effective for TAC but was most effective for the extraction of cyanidin-3-*O*-glucoside. Therefore, NaDES 7 was selected for the further analysis of the impact of process parameters on extraction efficiency and optimization.

### 2.2. Modelling and Optimization

The traditional “one-factor-at-a-time” extraction optimization approach involves performing a multitude of experimental trials and is materially and temporally consuming. These shortcomings can be overcome by employing innovative optimization approaches, such as RSM, as applied in this study. RSM allows for the decrease of the number of experiments necessary for model generation and optimization. Additionally, unlike traditional optimization methods, RSM enables the evaluation of interactions of independent variables [18].

#### 2.2.1. Model Fitting Assessment

Obtained experimental data for three independent variables (extraction temperature, time, and water amount in NaDES) and two response variables (cyanidin-3-*O*-glucoside and TAC) were included in the modeling and optimization (Table 2). The obtained values were fitted in a quadratic polynomial models and checked by ANOVA (Table 3). The remarkably significant quadratic polynomial model with a *p*-value less than 0.0001 and insignificant lack of fit with a *p*-value higher than 0.05 for both responses imply good efficiency of the developed models. The coefficients of variation that were lower than 10% indicate satisfactory response reproducibility. Additionally, the high values of the coefficients of determination (R^2^ = 0.9059 for cyanidin-3-*O*-glucoside and R^2^ = 0.9577 for TAC) confirm the good fit of the experimental values with the values predicted by the quadratic models.

#### 2.2.2. Influence of Extraction Parameters on Cyanidin-3-O-glucoside Extraction

The content of cyanidin-3-*O*-glycoside extracted from bilberry fruits ranged from a minimum of 0.14 mg/g DW (experiment 5: 20 °C, 60 min, 0% (*w*/*w*) water in NaDES) to a maximum of 0.28 mg/g DW (experiment 15: 50 °C, 60 min, 20% (*w*/*w*) water in NaDES). The broad range indicates a significant influence on process parameters on extraction efficiency and reasonableness for optimization.

According to the results of ANOVA (Table 3), the extraction temperature and water amount in NaDES (in linear, quadratic, and interaction terms) significantly affected the extraction efficiency of cyanidin-3-*O*-glucoside. The predictive equation of the accepted quadratic model for cyanidin-3-*O*-glucoside was developed after removing the attached term of negligible significance:

Y_cyanidin-3-*O*-glucoside_ (mg/g DW) = 0.2664 + 0.0121X_1_ + 0.0308X_3_ − 0.0224X_1_X_3_ − 0.0534X^2^_1_ − 0.0214X^2^_3_) (1)

**Figure 1:**
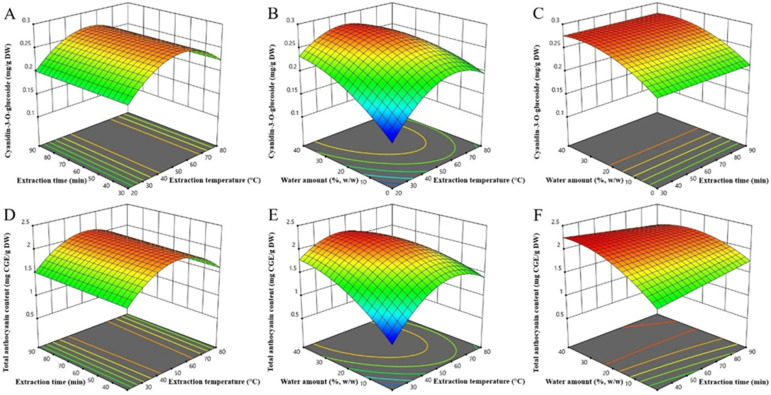
Response surfaces plots (3D) of cyanidin-3-*O*-glucoside (**A**–**C**) and total anthocyanins extracted (**D**–**F**) from bilberry fruits as a function of extraction temperature, time, and water amount in NaDES.

After generating an adequate model, the influence of the terms was analyzed and 3D surface plots (Figure 1) were used for that purpose. The positive effect of the extraction temperature (X_1_) and water content in the NaDES (X_2_) in linear terms confirms the importance of reducing the viscosity during extraction using the NaDES, bearing in mind that increasing both parameters contribute to efficiency in this way. However, an extreme increase in both parameters leads to the opposite effect, as evidenced by the negative quadratic terms. An excessive temperature causes the degradation of heat-sensitive anthocyanins, while the profuse water in the NaDES causes the breakdown of the hydrogen bond network.

The negative sign of X_1_X_3_ interaction suggests that the negative effect of temperature is more pronounced in conditions of higher water amount in the NaDES system. This effect can also be explained by the weakening of the hydrogen bond network due to the dilution of NaDES. At excessive water amounts, the complex supramolecular structure of NaDES is disturbed. Thus, the protective effect of NaDES disappears and anthocyanins are more susceptible to thermodegradation. Namely, there is a finding based on the NMR spectrum that NaDES-specific interactions are stable with the addition of water up to 50%. With further dilution, the NMR spectra are as aqueous solutions of the NaDES ingredients [19,20].

**Table 3:** Results of analysis of variance and quality of fitting for developed second-order polynomial models.

Source	*p*-Values	
	Cyanidin-3-*O*-glucoside	Total Anthocyanin Content
Linear terms		
X_1_—Extraction temperature	0.0433 *	0.0797
X_2_—Extraction time	/	0.6098
X_3_—Water amount	<0.0001 ***	<0.0001 ***
Quadratic terms		
X^2^_1_	<0.0001 ***	<0.0001 ***
X^2^_2_	/	/
X^2^_3_	0.0118 *	0.0013 **
Interaction terms		
X_1_X_2_	/	/
X_1_X_3_	0.0118 *	0.0008 ***
X_2_X_3_	/	0.0288 *
Model fitting assessment		
Model	<0.0001 ***	<0.0001 ***
Lack of fit	0.0755	0.1690
R^2^	0.9059	0.9577
Adjusted R^2^	0.8666	0.9281
Predicted R^2^	0.7477	0.8338
CV (%)	6.49	5.94

/—negligible significance (excluded from model); Level of significance—* 0.01 < *p* < 0.05; ** 0.001 < *p* < 0.01; *** *p* < 0.001.

#### 2.2.3. Influence of Extraction Parameters on TAC Extraction

The TAC in bilberry extracts differed from a minimum of 0.85 mg CGE/g DW (experiment 5: 20 °C, 60 min, 0% (*w*/*w*) water in NaDES) to a maximum of 2.22 mg CGE/g DW (experiment 11: 50 °C, 30 min, 40% (*w*/*w*) water in NaDES). Based on ANOVA (Table 3), the extraction efficiency of TAC was significantly influenced by the extraction time (quadratic term), the water amount in NaDES (linear and quadratic terms), their mutual interaction, as well as the interaction of the extraction time and the water amount in NaDES. The following model equation for TAC was developed by eliminating terms of negligible significance but keeping those required to support the hierarchy:

Y_TAC_ (mg CGE/g DW) = 2.10 + 0.0721X_1_ + 0.0195X_2_ + 0.2630X_3_ − 0.2488X_1_X_3_ − 0.1335X_2_X_3_ − 0.5280X²_1_ − 0.2213 X²_3_ (2)

A similar pattern of influence of temperature and water in NaDES (positive linear and negative quadratic and interaction terms) as in the above-mentioned cyanidin-3-*O*-glucoside model was reported. It is noteworthy that a significant negative effect of the interaction between the extraction time and the amount of water in NaDES was observed. It follows that prolonged UAE in diluted NaDES adversely affects the extraction efficiency of TAC. This may be related to the greater effect of cavitation in a less viscous medium, resulting in anthocyanin sonolysis if extraction takes a long time [13,17]. Prolonged sonication also leads to the accumulation of free radicals due to the sonochemistry effect, and anthocyanins as antioxidants are reduced to neutralize them [13]. The practical implication of this finding is that reducing the extraction time reduces energy consumption, which is significant from a “green chemistry” point of view. As today, minimizing energy consumption while keeping or improving the final chemical process output—i.e., maximizing energy intensity—has become a golden rule in green chemistry [21].

Overall, the extraction temperature showed the greatest influence on the extraction efficiency, followed by the amount of water in NaDES and the extraction time. Kumar et al. [22] showed that the increase in temperature and the percentage of added water significantly affects the viscosity of NADES, while Dai et al. [12] pointed out that in solvents of lower viscosity, the mobility of solutes is intensified, which also allows for better extraction. Due to such a significant effect of temperature on the extraction yield associated with thermal instability of anthocyanins, we decided to examine the stability of the optimized eutectic blueberry extract in storage conditions at different temperatures in the next stage of research.

#### 2.2.4. Optimization and Model Validation

Calculated optimal conditions in the examined experimental range to simultaneously obtain a maximum of both responses were a temperature of 48.38 °C, a time of 37.63 min, and 34.79% (*w*/*w*) water in NaDES (Table 4). The models were validated by preparing the extracts under defined optimal extraction conditions and comparing the experimentally obtained values with the predicted responses. Obtained extraction yields under optimal conditions were 0.27 ± 0.00 mg/g DW of cyanidin-3-*O*-glucoside and 2.12 ± 0.02 mg CGE/g DW of TAC. As expected, response values were included in the 95% confidence interval, which confirms the satisfactory predictive capacity of the developed RSM models.

### 2.3. Biological Activities

The application of bilberry phytocompounds as antimicrobial and antioxidant agents has been previously studied [23,24,25,26]. However, these studies were focused on conventionally obtained extracts while data on the activity of NaDES-based extracts are lacking.

The results of the antimicrobial activity of optimized bilberry extract against most common foodborne pathogens, skin pathogens, and causatives of cosmetic spoilage are shown in Table 5. Pure NaDES 7 used as solvent did not exhibit antimicrobial activity at the tested concentrations. On the other hand, the extract showed noteworthy activity against all tested bacteria and fungi. Gram-positive bacteria showed slightly higher sensitivity than Gram-negative bacteria to tested extract. The lowest minimal inhibitory concentration (MIC) of 15 mg/mL was noted for Gram-positive bacteria *Staphylococcus epidermidis* and *Listeria monocytogenes*. Similar concentrations were required to inhibit the growth of the yeast *Candida albicans* (MIC 15 mg/mL), while for the mold *Aspergillus brasiliensis* it was slightly higher (MIC 25 mg/mL). The high *Candida albicans* sensitivity is promising since this yeast is the cause of serious opportunistic infections of the skin and internal organs, such as the kidneys, heart, and brain. The most resistant among tested strains were Gram-negative bacteria *Pseudomonas aeruginosa* (MIC >35 mg/mL) and *Salmonella typhimurium* (MIC 35 mg/mL).

Similar to our findings, Zimmer et al., [27] reported that the most sensitive strains to blueberry extract were Gram-positive strains *Staphylococcus epidermidis*, *Streptococcus pyogenes*, *Proteus mirabilis*, and *Staphylococcus aureus.* Similarly, Silva et al. [28] have shown that blueberry extract has good antibacterial activity against *Staphylococcus aureus* and that extracts can inhibit biofilm formation and/or adhesion of *Pseudomonas aeruginosa, Escherichia coli, Proteus mirabilis*, *Acinetobacter baumannii*, and *Staphylococcus aureus.* Consistent with our findings, Cisovska et al. [29] showed that anthocyanins are broad-spectrum antimicrobial agents; however, Gram-positive bacteria are usually more sensitive than Gram-negative. According to these authors, the mechanisms underlying anthocyanin activity include both membrane and intracellular mediated activity. It is well known that differences in the cell wall structure between Gram-positive and Gram-negatives account for differences in the activity of many compounds. In Gram-negative bacteria, the outer cell membrane is fairly impermeable to large molecules and hampers the diffusion of hydrophobic compounds through the lipopolysaccharide layer [30,31]. Additionally, it has been shown that berry compounds can inhibit the growth of bacteria through multiple mechanisms, including the destabilization and permeabilization of the cytoplasmic membrane, inhibition of extracellular microbial enzymes, interfering with cellular microbial metabolism, and deprivation of essential substrates. Additionally, berry compounds can affect the anti-adherence of bacteria to epithelial cells as a prerequisite for the colonization of many pathogens [23]. According to Ma et al. [32], anthocyanins and catechins are good growth inhibitors of various microorganisms and offer a promising new approach to preventing foodborne disease. They found that anthocyanins can significantly reduce the abundance of pathogenic bacteria that produce toxins in the host, including *Desulfovibrio* sp. and *Enterococcus*, and can increase the abundance of probiotics, such as *Akkermansia* and *Bifidobacteria*. Finally, it seems that anthocyanins and catechins can regulate the composition of intestinal microbes to improve intestinal immunity and promote intestinal health, thereby controlling foodborne disease.

Antioxidant activity was considered by determining the radical scavenging activity. The radical scavenging activity for the optimized extract was calculated as a percentage of DPPH inhibition and was 85.60 ± 0.41%. The antioxidant activity of pure NaDES 7 used as solvent was determined in the same way and was negligibly low. This implied that there was no significant coaction with the NaDES constituents, but rather that the radical scavenging activity came from the bilberry phytochemicals. Bilberry anthocyanins are well-known agents effective against oxidative stress. By this action mechanism, anthocyanins prevent and cure various diseases, such as cardiovascular diseases, diabetes, macular degeneration, anticancer, and the protective effect against radio- and chemotherapy [26]. Beyond anthocyanins, other polyphenolics that were not covered by this study almost certainly contributed to radical scavenging activities.

### 2.4. Storage Stability

Since anthocyanins are susceptible to degradation, it is crucial to consider the long-term stability under conditions simulating the real storage environment for the potential commercial application of the extract. Therefore, the storage stability of the previously optimized bilberry fruits extract was tested under different temperatures (4, 25, and 40 °C) for 30 days of keeping in the dark. For practical reasons, the influence of natural light indoors at normal room temperature of 25 °C was also examined.

**Figure 2:**
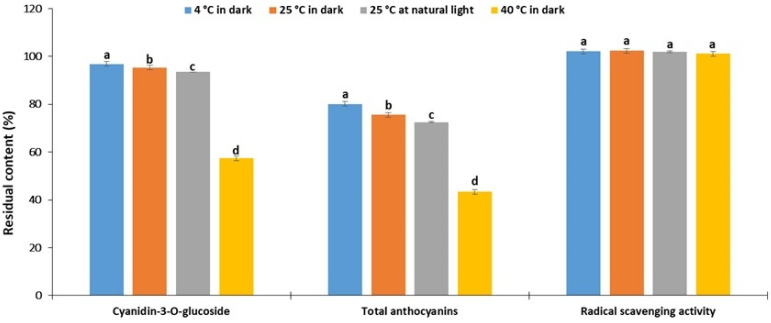
The residual content of anthocyanins and radical scavenging (DPPH) activity after 30 days of storage under different conditions. a–d—the different letters above the bars represent a significant difference in residual contents between samples stored at the various condition with a significance level of 0.05 according to ANOVA with Tukey’s post hoc test.

As can be observed in Figure 2, the residual contents of cyanidin-3-*O*-glucoside and TAC decreased with increased temperature. This is consistent with the results of stability studies on blueberry pomace anthocyanins extracted using choline chloride–oxalic acid [13] and black carrot anthocyanins extracted using choline chloride–citric acid [14]. It is noteworthy that the difference in residual contents between the samples stored at 4 °C and 25 °C was negligible (less than 10%), while this difference compared to samples stored at 40 °C was dramatic. A comparison of storage at 25 °C in the dark and natural light environments also shows a negligible difference in the residual content of anthocyanins (about 2% for cyanidin-3-*O*-glucoside and 3% for TAC), suggesting satisfactory protection against light-induced degradation. Similar conclusions were reached in the above-mentioned stability study on blueberry anthocyanins [13]. 

Contrary to expectations, the DPPH radical scavenging activity remained preserved during storage under all tested conditions (Figure 2). This finding can be partially explained by the fact that the degradation of anthocyanins leads to the formation of phenolic acids (such as gallic and protocatechuic acids), which also showed radical scavenging activity [33]. To fully address this assumption, further experiments are required. These results also indicate that the interpretation of results based on DPPH activity alone may lead to a false positive conclusion. Overall, the results of the current study unequivocally show that refrigerated storage at 4 °C in dark is most suitable for preserving bilberry anthocyanins.

The stabilizing effect of polyphenolic compounds in the NaDES system can be related to their ability to participate in intermolecular interactions with NaDES components, particularly hydrogen bonding interactions. Hydrogen bonding interactions reduce the mobility of solute molecules. This reduces exposure to oxygen at the solvent-air interface, thereby reducing oxidative degradation as the main mechanism of polyphenolic degradation [12]. This hypothesis is consistent with previous findings, such as the greater stability of bilberry anthocyanins obtained by enzyme-assisted extraction compared to conventional extraction due to increased interactions with enzymatically modified pectin molecules [4].

## 3. Materials and Methods

### 3.1. Plant Material

Fully ripe bilberry fruits were purchased at a local store in Belgrade (Serbia). The fruits were dried in a laboratory dryer for 3 days (d) at 40 °C to humidity of 3.51 ± 0.25%. The dried fruits were crushed in a laboratory mill and sieved through a set of sieves prescribed by the Yugoslav Pharmacopoeia 2000 [34]. Milled bilberry fruits of the particle size from 0.75 to 2 mm were used further for extraction.

### 3.2. Screening Analysis of NaDES

In the first stage of the study, a screening analysis of ten different NaDES was performed to select the most suitable one for the extraction of anthocyanins from bilberry fruits.

#### 3.2.1. Preparation of NaDES

All examined NaDES were prepared using the heating and stirring method. Solvent components were weighed into glass bottles in the molar proportions shown in Table 1. The sealed bottles were heated to 80 °C with constant stirring (120 rpm) until a homogeneous transparent liquid was formed.

#### 3.2.2. Extraction Procedure

The extraction of bilberry fruit anthocyanins using NaDES as solvents was performed using the solid–liquid ultrasound-assisted extraction (UAE) method. All extraction procedures were carried out in a Bandelin Sonorex (Germany) ultrasonic water bath at a constant power of 320 W and frequency of 35 kHz. The plant samples (1 g) were extracted in sealed conical Falcon test tubes with 10 mL of solvent. During the screening analysis, the extraction conditions were set constant (sonication time 60 min, temperature 50 °C, liquid to solid ratio 10 mL/g). To reduce viscosity, all NaDES were diluted with 20% *w*/*w* deionized water before screening analysis. The extraction efficiency of NaDES was compared with methanol as conventional solvents. All experiments were performed in triplicate. The solid–liquid extraction mixture was filtered through the Whatman N°1 filter paper and chemically analyzed immediately after the end of the extraction.

### 3.3. Optimization by RSM

In the next step of the study, the optimization of the extraction process with the previously selected solvent was performed employing RSM. The extraction temperature (X_1_), extraction time (X_2_), and water content in NaDES (X_3_) were analyzed as independent variables. The extraction efficiency of dominant anthocyanin compound cyanidin-3-*O*-glucoside and total anthocyanins (TAC) were monitored as response (dependent) variables. In accordance with the Box–Behnken experimental design (BBD), the independent variables were varied at three levels coded with −1, 0, and +1. The BBD scheme of 18 single experiments, including 6 in the central points, with independent variables in the form of coded and actual values, is shown in Table 2.

The obtained experimental values were fitted to the second-order polynomial model equation:

(3) $$Y=\beta_{0}+\overset{3}{\underset{i=1}{\sum }}\beta_{i}X_{i}+\overset{3}{\underset{i=1}{\sum }}\beta_{ii}X_{i}^{2}+\overset{3}{\underset{i<j=1}{\sum }}\beta_{ij}X_{i}X_{j}$$

Symbol *Y* represents the predicted response variable; X*_i_* and X*_j_* represent independent variables; while β_0_, β*_i_*, β*_ii_*, and β*_ij_* represent regression coefficients (intercept, linearity, quadratic, and interaction, respectively). The adequacy of models to meet the experimentally obtained data was tested by ANOVA with a 0.05 level of significance. Model adequacy was evaluated by considering the model, lack-of-fit testing, the coefficient of determination, and the coefficient of variation.

The optimization aimed to maximize the extraction efficiency of cyanidin-3-*O*-glucosides and TAC simultaneously. Optimal extraction conditions were calculated using the desirability function. The validity of the optimized models was confirmed by test extraction under calculated optimal extraction conditions in triplicate and comparing the predicted and achieved values of extraction efficiency. The optimization process was performed using Design Expert Trial software, version 11 (Stat-Ease, Minneapolis, MN, USA).

The further investigations of biological activities and storage stability were performed with the optimized extract in liquid form.

### 3.4. Chemical Analysis

#### 3.4.1. HPLC Analysis

The quantification of the dominant anthocyanin compound cyanidin-3-*O*-glucoside was performed by HPLC analysis according to the procedure described in European Pharmacopoeia 10.0 (2019) (monograph 04/2019:02394) with slight modifications [35]. All extract samples were diluted with deionized water (1:1 *v*/*v*) immediately before chromatographic analysis and filtered using a 0.45 μL syringe filter. An Agilent series 1200 HPLC with a diode array detector and a reverse phase analytical column Lichrospher RP-18 (250 × 4 mm i.d., 5 µm particle size, Agilent) were used. The mobile phase was composed of solution A (anhydrous formic acid and water, 8.5:91.5 *v*/*v*) and solution B (anhydrous formic acid, acetonitrile, methanol, and water, 8.5:22.5:22.5:41.5 *v*/*v*/*v*/*v*). The sample was injected at a volume of 20 µL and separated at a flow rate of 1 mL/min by the following elution program: starting with the solution B 7%, from 7% to 25% on 35 min, from 25% to 65% on 45 min, and from 65% to 100% on 46 min and maintained during 46–50 min. The column was thermostated at 30 °C and the detection wavelength was set at 535 nm. The cyanidin-3-*O*-glucoside peak was identified based on the position in the chromatogram and the UV spectrum of the authentic standard. Its amount was determined using the calibration curve of the standard and is expressed as mg per g of dried drug weight (mg/g DW).

#### 3.4.2. Quantification of TAC

The TAC was quantified spectrophotometrically as described in European Pharmacopoeia 10.0 (2019) (monograph 01/2019:1602) with slight modifications [35]. The extract absorbance was recorded at 528 nm using acidified methanol (0.1% *v*/*v* hydrochloric acid) as a compensating solution. All determinations were carried out in triplicate. Results are presented as mean and expressed as mg of cyanidin-3-*O*-glucoside equivalent per g of dried drug weight (mg CGE/g DW).

### 3.5. Biological Activities

#### 3.5.1. Antimicrobial Activity

The optimized bilberry extract was tested against foodborne bacterial pathogens (*Escherichia coli* O157:H7, *Salmonella typhimurium* ATCC 14028, *Shigella flexneri* ATCC 12022, *Listeria monocytogenes* ATCC 19114, and *Enterococcus faecalis* ATCC 29212), as well as microorganisms related with skin infections and spoilage of cosmetic products (bacteria *Escherichia coli* ATCC 8739, *Pseudomonas aeruginosa* ATCC 27853, *Staphylococcus epidermidis* ATCC 12228, *Staphylococcus aureus* ATCC 25923; the yeast *Candida albicans* ATCC 10231, and mold *Aspergillus brasiliensis* ATCC 16404).

The antimicrobial activity was screened using the broth microdilution method, performed according to the recommendations of the National Committee for Clinical Laboratory Standards (CLSI) [36]. Bacteria were sub-cultured on Mueller Hinton Agar (MHA) (pH 7.2) and incubated for 24 h at 37 °C. Bacterial strains were cultured overnight in Mueller Hinton Broth (MHB) medium at 37 °C. The bacterial culture was adjusted to an optical density equivalent to 1 × 10^8^ CFU/mL. Bacterial cell suspensions were centrifuged for 10 min at 4000 rpm and then resuspended with 0.01 M MgSO_4_ to a final concentration of 10^6^ CFU/ml. For fungi, inocula were prepared by growing on Sabouraud Dextrose Agar (SAB) for *C. albicans* and Potato Dextrose Agar for *A. brasiliensis*. Slopes were flooded with 0.85% saline and conidia were gently probed. The resulting suspensions were removed and vortexed thoroughly. After the settling of the larger particles, suspensions were adjusted by nephelometry and diluted in saline to obtain inocula of 2 × 10^4^ CFU/mL, as confirmed by colony counts in triplicate on SAB agar.

The serial doubling dilutions of the extract were prepared in 96-well microtiter trays in the appropriate medium (MHB for bacteria and Tryptic Soy Broth for fungi). After the addition of the inoculum of the microorganism, the trays were incubated for 24 h at 37 °C in case of bacteria and 3–7 d at 25 °C in case of fungi. The antimicrobial activity was determined as minimum inhibitory concentrations (MIC) and minimum bactericidal/fungicidal concentration (MBC/MFC). Bacterial cell viability was determined by the resazurin reaction. The resazurin reaction was monitored by a change in the color from purple to pink. The indicator resazurin in the presence of living bacterial cells was reduced to pink resofurin, which is an indication of cell growth. The lowest concentration at which there was no change in the color of resazurin was interpreted as the MIC. MBC was determined by re-inoculating 2 µL from wells in which there was no growth (MIC concentration and higher tested) in 100 µL of sterile liquid medium and re-incubating at the appropriate temperature. The lowest concentration at which there was no growth was declared as the MBC. On the other hand, MFC was determined by spot inoculating (10 μL) from wells not visibly turbid on SAB agar plates and that were incubated for 3 d at 25 °C. MFC was defined as the lowest concentration resulting in no growth on the subculture. All tests were performed in triplicate.

#### 3.5.2. Radical Scavenging Activity

The radical scavenging activity was evaluated for the optimized extract and was also used as a marker in the stability study (as described in Section 3.6). Bearing in mind the literature data that the NaDES themselves can exhibit biological activities, the radical scavenging activity of the pure NaDES 7 was also evaluated. The radical scavenging activity was measured using the 2,2-diphenyl-1-picrylhydrazyl (DPPH) assay described by Blois [37]. Briefly, a solution of DPPH in methanol (900 μL) and a liquid extract sample (100 μL) were vigorously mixed and incubated at room temperature in a dark place for 20 min. Absorbance was measured at 517 nm. Methanol was used as a negative control. Activities are expressed as a percentage of DPPH radical inhibition as follows:

DPPH inhibition (%) = [(A_C_ − A_S_)/A_C_] × 100, (4)

where A_C_ represents the absorbance of the control and A_S_ the absorbance of the sample. All measurements were made in triplicates and the results were expressed as mean value ± standard deviation.

### 3.6. Storage Stability

To determine the storage stability, samples of bilberry extract obtained using previously selected NaDES under optimized extraction conditions were transferred into sealed clear glass vials. Sample vials were stored for 30 d at 4, 25, and 40 °C in a dark environment (vials wrapped in aluminum foil), as well as at 25 °C exposed to natural light indoors (transparent glass vials without aluminum foil). Stability under varying storage conditions was assessed by comparing the retention of cyanidin-3-*O*-glucoside, TAC, and DPPH radical scavenging activity. The residual content was calculated as the ratio of compound content/activity on the last and first day (optimized extract) of the study expressed as a percentage.

### 3.7. Statistical Analysis

The extraction yields of target compounds in screening analysis, biological activities, and storage stability of multiple groups were compared using one-way ANOVA followed by Tukey’s post hoc test. Mean values were considered statistically different if the *p*-values were lower than 0.05. Statistical data processing was performed using SPPS Trial software (Dublin, Ireland).

## 4. Conclusions

The presented study considered the potential application of NaDES integrated with UAE for the extraction of anthocyanins from bilberry fruits. Among the ten different choline chloride-based NaDES, choline chloride:sorbitol (1:1) was the most efficient. This solvent was chosen for the next stage of research, which included analyzing the impact of extraction conditions (extraction time, temperature, and amount of water in NaDES) and their optimization using RSM. The extraction yields recorded under the optimized extraction conditions (37.63 min, 48.38 °C, and 34.79% (*w*/*w*) water in NaDES) were 0.2751 mg/g DW of cyanidin-3-*O*-glucoside and 2.12 mg CGE/g DW of TAC. The optimized extract showed promising DPPH radical scavenging activity as well as antimicrobial activity against most common foodborne pathogens, skin pathogens, and causatives of cosmetic spoilage. A 30-day stability study of the optimized extract showed that refrigerated storage at 4 °C in dark is most suitable for preserving bilberry anthocyanins. An interesting finding is that with increasing storage temperature, the content of cyanidin-3-*O*-glucoside and TAC decreased, while the DPPH radical scavenging activity remained constant. Overall, NaDES–UAE as an innovative, sustainable, and eco-friendly method could be an effective alternative to conventional anthocyanins extraction techniques with considerable commercial potential in the pharmaceutical, cosmetic, and food industries.

## Figures and Tables

**Table 1:** List of natural deep eutectic solvents estimated in initial screening analysis for the extraction of total anthocyanins and cyanidin-3-*O*-glucoside from bilberry fruits.

Solvent *	Composition	Molar Ratio	Cyanidin-3-*O*-glucoside (mg/g DW)	Total Anthocyanin Content (mg CGE/g DW)
NaDES 1	Choline chloride:Lactic acid	1:2	0.19 ± 0.01 ^d^	2.48 ± 0.02 ^b^
NaDES 2	Choline chloride:Citric acid:Water	1:1:2	0.28 ± 0.00 ^a^	1.78 ± 0.04 ^f^
NaDES 3	Choline chloride:Malic acid:Water	1:1:2	0.28 ± 0.01 ^a^	2.24 ± 0.06 ^c^
NaDES 4	Choline chloride:Tartaric acid	1:2	0.20 ± 0.00 ^cd^	1.25 ± 0.04 ^h^
NaDES 5	Choline chloride:Glycerol	1:2	0.28 ± 0.01 ^a^	1.90 ± 0.04 ^e^
NaDES 6	Choline chloride:1,2-propanediol	1:3	0.21 ± 0.00 ^c^	2.00 ± 0.02 ^de^
NaDES 7	Choline chloride:Sorbitol	1:1	0.29 ± 0.00 ^a^	2.03 ± 0.01 ^d^
NaDES 8	Choline chloride:Glucose:Water	2:1:1	0.29 ± 0.01 ^a^	1.74 ± 0.06 ^fg^
NaDES 9	Choline chloride:Fructose:Water	2:1:1	0.25 ± 0.00 ^b^	1.64 ± 0.06 ^g^
NaDES 10	Choline chloride:Urea	1:2	0.20 ± 0.01 ^cd^	0.92 ± 0.01 ^i^
MeOH	Methanol		0.20 ± 0.00 ^cd^	2.93 ± 0.01 ^a^

* To reduce viscosity, NaDES 1–10 were diluted with deionized water (20% *w*/*w*) before screening analysis. Mean values in column followed by different superscript letters (a–i) are significantly different (*p* < 0.05) according to ANOVA with Tukey’s post hoc test; mg CGE/g DW—mg cyanidin-3-*O*-glucoside equivalents per g dry weight of the plant sample.

**Table 2:** Total anthocyanins and cyanidin-3-*O*-glucoside from bilberry fruits extracted under different conditions defined according to the Box–Behnken experimental design.

Experiment	Factor 1	Factor 2	Factor 3	Response 1	Response 2
	X_1_: Extraction Temperature (°C)	X_2_: Extraction Time (min)	X_3_: Water Amount (%, *w*/*w*)	Cyanidin-3-*O*-glucoside (mg/g DW)	Total Anthocyanin Content (mg CGE/g DW)
1	20 (−1)	30 (−1)	20 (0)	0.19	1.33
2	80 (+1)	30 (−1)	20 (0)	0.24	1.78
3	20 (−1)	90 (+1)	20 (0)	0.19	1.50
4	80 (+1)	90 (+1)	20 (0)	0.21	1.57
5	20 (−1)	60 (0)	0 (−1)	0.14	0.85
6	80 (+1)	60 (0)	0 (−1)	0.20	1.38
7	20 (−1)	60 (0)	40 (+1)	0.24	1.85
8	80 (+1)	60 (0)	40 (+1)	0.21	1.38
9	50 (0)	30 (−1)	0 (−1)	0.18	1.39
10	50 (0)	90 (+1)	0 (−1)	0.23	1.76
11	50 (0)	30 (−1)	40 (+1)	0.28	2.22
12	50 (0)	90 (+1)	40 (+1)	0.27	2.05
13	50 (0)	60 (0)	20 (0)	0.27	2.06
14	50 (0)	60 (0)	20 (0)	0.28	2.22
15	50 (0)	60 (0)	20 (0)	0.28	2.19
16	50 (0)	60 (0)	20 (0)	0.26	2.10
17	50 (0)	60 (0)	20 (0)	0.26	2.07
18	50 (0)	60 (0)	20 (0)	0.26	2.02

mg CGE/g DW—mg cyanidin-3-*O*-glucoside equivalents per g dry weight of the plant sample.

**Table 4:** Comparison of predicted and experimentally obtained extraction yields of monitored responses under determined optimal extraction conditions.

Optimized Extraction Conditions	Responses under Optimized Extraction Conditions		
Extraction temperature: 48.38 °C Extraction time: 37.63 min Water amount: 34.79% (*w*/*w*) (Desirability: 0.98)	Target compounds	Predicted mean (95% confidence interval)	Validated values (mean ± standard deviation)
	Cyanidin-3-*O*-glucoside (mg/g DW)	0.28 (0.26–0.29)	0.27 ± 0.00
	Total anthocyanin content (mg CGE/g DW)	2.23 (2.10–2.36)	2.12 ± 0.02

mg CGE/g DW—mg cyanidin-3-*O*-glucoside equivalents per g dry weight of the plant sample.

**Table 5:** Antimicrobial activity of optimized bilberry extract.

Microorganism		MIC (mg/mL)	MBC/MFC (mg/mL)
Foodborne pathogens	*Escherichia coli* O157:H7	20	25
	*Salmonella typhimurium* ATCC 14028	35	>35
	*Shigella flexneri* ATCC 12022	25	30
	*Listeria monocytogenes* ATCC 19114	15	20
	*Enterococcus faecalis* ATCC 29212	25	25
Causatives of skin infections and cosmetic products spoilage	*Escherichia coli* ATCC 8739	25	30
	*Pseudomonas aeruginosa* ATCC 27853	>35	>35
	*Staphylococcus aureus* ATCC 25923	15	20
	*Staphylococcus epidermidis* ATCC 12228	15	15
	*Candida albicans* ATCC 10231	15	20
	*Aspergillus brasiliensis* ATCC 16404	25	30

MIC—minimum inhibitory concentration; MBC/MFC—minimum bactericidal/fungicidal concentration.

## Data Availability

The data that support the findings of this study are available on request from the corresponding author.

## References

[1] Dare A.P., Günther C.S., Grey A.C., Guo G., Demarais N.J., Cordiner S., McGhie T.K., Boldingh H., Hunt M., Deng C. (2022). Resolving the developmental distribution patterns of polyphenols and related primary metabolites in bilberry (*Vaccinium myrtillus*) fruit. Food Chem..

[2] (2014). ESCOP Monographs: The Scientific Foundation for Herbal Medicinal Products.

[3] Primetta A.K., Jaakola L., Ayaz F.A., Inceer H., Riihinen K.R. (2013). Anthocyanin fingerprinting for authenticity studies of bilberry (*Vaccinium myrtillus* L.). Food Control.

[4] Dinkova R., Heffels P., Shikov V., Weber F., Schieber A., Mihalev K. (2014). Effect of enzyme-assisted extraction on the chilled storage stability of bilberry (*Vaccinium myrtillus* L.) anthocyanins in skin extracts and freshly pressed juices. Food Res. Int..

[5] Da Silva D.T., Pauletto R., Da Silva Cavalheiro S., Bochi V.C., Rodrigues E., Weber J., Emanuelli T. (2020). Natural deep eutectic solvents as a biocompatible tool for the extraction of blueberry anthocyanins. J. Food Compost. Anal..

[6] Klimaviciute R., Navikaite V., Jakstas V., Ivanauskas L. (2015). Complexes of dextran sulfate and anthocyanins from *Vaccinium myrtillus*: Formation and stability. Carbohydr. Polym..

[7] Enaru B., Drețcanu G., Pop T.D., Stǎnilǎ A., Diaconeasa Z. (2021). Anthocyanins: Factors Affecting Their Stability and Degradation. Antioxidants.

[8] Ferreira L.F., Minuzzi N.M., Rodrigues R.F., Pauletto R., Rodrigues E., Emanuelli T., Bochi V.C. (2020). Citric acid water-based solution for blueberry bagasse anthocyanins recovery: Optimization and comparisons with microwave-assisted extraction (MAE). LWT.

[9] Mohd Fuad F., Mohd Nadzir M., Kamaruddin H.A. (2021). Hydrophilic natural deep eutectic solvent: A review on physicochemical properties and extractability of bioactive compounds. J. Mol. Liq..

[10] Xing C., Cui W.Q., Zhang Y., Zou X.S., Hao J.Y., Zheng S.D., Wang T.T., Wang X.Z., Wu T., Liu Y.Y. (2022). Ultrasound-assisted deep eutectic solvents extraction of glabridin and isoliquiritigenin from *Glycyrrhiza glabra*: Optimization, extraction mechanism and in vitro bioactivities. Ultrason. Sonochem..

[11] Santana A.P.R., Mora-Vargas J.A., Guimarães T.G.S., Amaral C.D.B., Oliveira A., Gonzalez M.H. (2019). Sustainable synthesis of natural deep eutectic solvents (NADES) by different methods. J. Mol. Liq..

[12] Dai Y., Rozema E., Verpoorte R., Choi Y.H. (2016). Application of natural deep eutectic solvents to the extraction of anthocyanins from *Catharanthus roseus* with high extractability and stability replacing conventional organic solvents. J. Chromatogr. A.

[13] Fu X., Wang D., Belwal T., Xie J., Xu Y., Li L., Luo Z. (2021). Natural deep eutectic solvent enhanced pulse-ultrasonication assisted extraction as a multi-stability protective and efficient green strategy to extract anthocyanin from blueberry pomace. LWT.

[14] Türker D.A., Doğan M. (2022). Ultrasound-assisted natural deep eutectic solvent extraction of anthocyanin from black carrots: Optimization, cytotoxicity, in-vitro bioavailability and stability. Food Bioprod. Process..

[15] Da Silva D.T., Smaniotto F.A., Costa I.F., Baranzelli J., Muller A., Somacal S., Emanuelli T. (2021). Natural deep eutectic solvent (NADES): A strategy to improve the bioavailability of blueberry phenolic compounds in a ready-to-use extract. Food Chem..

[16] Jovanović M., Mudrić J., Drinić Z., Matejić J., Kitić D., Bigović D., Šavikin K. (2022). Optimization of ultrasound-assisted extraction of bitter compounds and polyphenols from willow gentian underground parts. Sep. Purif. Technol..

[17] Rae J., Ashokkumar M., Eulaerts O., Von Sonntag C., Reisse J., Grieser F. (2005). Estimation of ultrasound induced cavitation bubble temperatures in aqueous solutions. Ultrason. Sonochem..

[18] Kumar K., Srivastav S., Sharanagat V.S. (2020). Ultrasound Assisted Extraction (UAE) of Bioactive Compounds from Fruit and Vegetable Processing By-Products: A Review. Ultrason. Sonochem..

[19] Dai Y., Varypataki E.M., Golovina E.A., Jiskoot W., Witkamp G.J., Choi Y.H., Verpoorte R. (2020). Natural deep eutectic solvents in plants and plant cells: In vitro evidence for their possible functions. Advances in Botanical Research.

[20] Gómez A.V., Tadini C.C., Biswas A., Buttrum M., Kim S., Boddu V.M., Cheng H.N. (2019). Microwave-assisted extraction of soluble sugars from banana puree with natural deep eutectic solvents (NADES). LWT.

[21] Quadrelli E.A. (2016). 25 years of energy and green chemistry: Saving, storing, distributing and using energy responsibly. Green Chem..

[22] Kumar A.K., Parikh B.S., Pravakar M. (2016). Natural deep eutectic solvent mediated pretreatment of rice straw: Bioanalytical characterization of lignin extract and enzymatic hydrolysis of pretreated biomass residue. Environ. Sci. Pollut. Res. Int..

[23] Puupponen-Pimiä R., Nohynek L., Alakomi H.L., Oksman-Caldentey K.M. (2005). The action of berry phenolics against human intestinal pathogens. BioFactors.

[24] Klavins L., Mezulis M., Nikolajeva V., Klavins M. (2021). Composition, sun protective and antimicrobial activity of lipophilic bilberry (*Vaccinium myrtillus* L.) and lingonberry (*Vaccinium vitis-idaea* L.) extract fractions. LWT.

[25] Satoh Y., Ishihara K. (2020). Investigation of the antimicrobial activity of Bilberry (*Vaccinium myrtillus* L.) extract against periodontopathic bacteria. J. Oral Biosci..

[26] Thornthwaite J.T., Thibado S.P., Thornthwaite K.A. (2020). Bilberry anthocyanins as agents to address oxidative stress. Pathology, Oxidative Stress and Dietary Antioxidants.

[27] Zimmer R., Blum-Silva H., Kulkamp Souza L., WulffSchuch M., Reginatto H., Pereira P., Lencina L. (2014). The antibiofilm effect of blueberry fruit cultivars against *Staphylococcus epidermidis* and *Pseudomonas aeruginosa*. J. Med. Food.

[28] Silva S., Costa M., Mendes M., Morais M., Calhau C., Pintado P. (2016). Antimicrobial, antiadhesive and antibiofilm activity of an ethanolic, anthocyanin-rich blueberry extract purified by solid phase extraction. J. Appl. Microbiol..

[29] Cisowska A., Wojnicz D., Hendrich A. (2011). Anthocyanins as Antimicrobial Agents of Natural Plant Origin. Nat. Prod. Commun..

[30] Tavares D., Antunes C., Padrão J., Ribeiro I., Zille A., Amorim P., Ferreira F., Felgueiras P. (2020). Activity of Specialized Biomolecules against Gram-Positive and Gram-Negative Bacteria. Antibiotics.

[31] Reygaert W.C. (2018). An overview of the antimicrobial resistance mechanisms of bacteria. AIMS Microbiol..

[32] Ma Y., Ding S., Fei Y., Liu G., Jang H., Fang J. (2019). Antimicrobial activity of anthocyanins and catechins against foodborne pathogens *Escherichia coli* and *Salmonella*. Food Control.

[33] Sinela A., Rawat N., Mertz C., Achir N., Fulcrand H., Dornier M. (2017). Anthocyanins degradation during storage of *Hibiscus sabdariffa* extract and evolution of its degradation products. Food Chem..

[34] (2000). Yugoslavian Pharmacopeia (Pharmacopoea Jugoslavica).

[35] (2019). European Pharmacopoeia 10.0.

[36] (2002). National Committee for Clinical Laboratory Standards (CLSI) Reference Method for Broth Dilution Antifungal Susceptibility Testing of Filamentous Fungi.

[37] Blois M.S. (1958). Antioxidant Determinations by the Use of a Stable Free Radical. Nature.

